# Higher Fetal Insulin Resistance in Chinese Pregnant Women with Gestational Diabetes Mellitus and Correlation with Maternal Insulin Resistance

**DOI:** 10.1371/journal.pone.0059845

**Published:** 2013-04-01

**Authors:** Qiuwei Wang, Ruiping Huang, Bin Yu, Fang Cao, Huiyan Wang, Ming Zhang, Xinhong Wang, Bin Zhang, Hong Zhou, Ziqiang Zhu

**Affiliations:** Changzhou Women and Children Health Hospital Affiliated to Nanjing Medical University, Changzhou, Jiangsu Province, China; Virgen Macarena University Hospital, School of Medicine, Spain

## Abstract

**Objective:**

The aim of this study was to determine the effect of gestational diabetes mellitus (GDM) on fetal insulin resistance or β-cell function in Chinese pregnant women with GDM.

**Measurements:**

Maternal fasting blood and venous cord blood samples (reflecting fetal condition) were collected in 65 well-controlled Chinese GDM mothers (only given dietary intervention) and 83 control subjects. The insulin, glucose and proinsulin concentrations of both maternal and cord blood samples were measured, and the homeostasis model assessment of insulin resistance (HOMA-IR) and the proinsulin-to-insulin ratios (an indicator of fetal β-cell function) were calculated in maternal and cord blood respectively.

**Results:**

Both maternal and fetal levels of insulin, proinsulin and HOMA-IR but not proinsulin-to-insulin ratios were significantly higher in the GDM group than in the control group (maternal insulin, 24.8 vs. 15.4 µU/mL, *P* = 0.004, proinsulin, 23.3 vs. 16.2 pmol/L, *P* = 0.005, and HOMA-IR, 5.5 vs. 3.5, *P* = 0.041, respectively; fetal: insulin, 15.1 vs. 7.9 µU/mL, *P*<0.001, proinsulin, 25.8 vs. 15.1 pmol/L, *P* = 0.015, and HOMA-IR, 2.8 vs. 1.4, *P* = 0.017, respectively). Fetal HOMA-IR but not proinsulin-to-insulin ratios was significantly correlated to maternal HOMA-IR (r = 0.307, *P* = 0.019), in the pregnant women with GDM.

**Conclusions:**

Fetal insulin resistance was higher in Chinese pregnant women with GDM than control subjects, and correlated with maternal insulin resistance.

## Introduction

The rapid and significant increase in the incidence of metabolic syndrome and type II diabetes has become a worldwide concern in both developed and developing countries [1], [2]. There are strong evidential supports for the “fetal origins” hypothesis, which connects adult metabolic syndrome to adverse intrauterine conditions and related disproportionate fetal growth [3]–[5], especially in the type II diabetes [6]–[14].

A great deal of research has revealed the pivotal role of insulin resistance and β-cell function in the development of type II diabetes in adults. Offspring of mothers with insulin resistance (e.g. GDM or obese mothers) are far more likely to develop metabolic syndrome and type II diabetes [9, 10, and 15]. However, only a few latest studies focused on the effect of GDM on fetal levels of insulin resistance or β-cell function in pregnant women with GDM. In 2009, it was firstly reported that fetuses of obese mothers (who had insulin resistance) developed insulin resistance in utero [16]. Furthermore, in 2010, another study reported that oral glucose tolerance test (OGTT) blood glucose concentrations were strongly negatively associated with decreased fetal insulin sensitivity in pregnant women with GDM [17]. Similarly, our study was to determine the effect of GDM on fetal insulin resistance or β-cell function in Chinese pregnant women with GDM. Such related research may be help to find a potential effective intervention during pregnancy to halt the increasing epidemic of metabolic syndromes and type II diabetes.

## Subjects and Methods

### Ethics Statement

This study was approved by Changzhou Health Bureau and the ethics committee of Changzhou Women and Children Health Hospital affiliated to Nanjing Medical University (No. ZD201013). The written informed consent was obtained from each subject in this manuscript.

### Study Subjects

**Figure 1:**
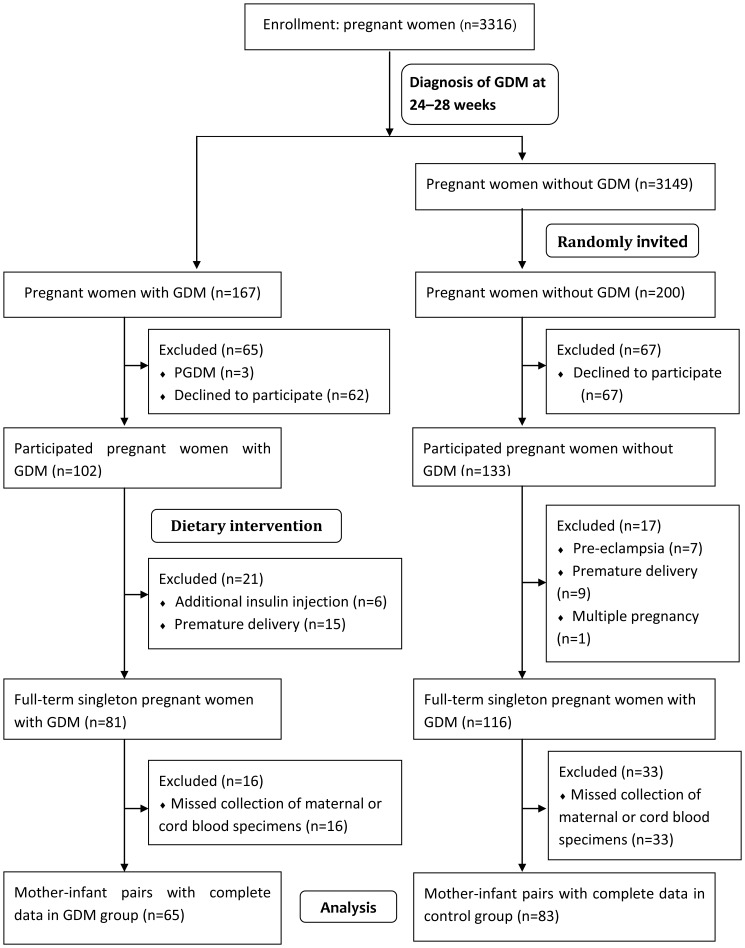
Flow diagram. GDM, gestational diabetes mellitus; PGDM, pregestational diabetes mellitus.

This was a prospective maternal-fetal case-controlled cohort study. Pregnant women were recruited from Changzhou Women and Children Health Hospital affiliated to Nanjing Medical University from March 2010 to June 2011, as shown in figure 1.

167 GDM and 3149 control subjects were included as candidates in this study, on the basis of a diagnosis of GDM at 24–28 weeks of gestation. 200 subjects were randomly invited in the control group. 62 GDM and 67 control subjects declined to participate this study, and 3 pregnant women with pregestational diabetes mellitus was not invited in the GDM group. 102 GDM and 133 control subjects participated in this study. The pregnant women with GDM were well managed by dietary intervention and blood glucose monitoring for achieving euglycemia. Dietary intervention included low-carbohydrate and low-fat diets intake, as well as optimizing of dietary fatty acid composition. In the GDM group, 21 were excluded as 6 for additional insulin injection, and 15 for premature delivery. In the control group, 17 were excluded, as 7 for pre-eclampsia, 9 for premature delivery, and 1 for a multiple pregnancy. 16 GDM and 33 control subjects were excluded, as we missed collection of their maternal blood or cord blood specimens. 65 GDM and 83 control mother-infant pairs (total 75.1% of eligible participants) constituted our final study cohort, with complete data on all studied biomarkers in maternal and cord blood specimens. There were no significant differences in maternal characteristics between patients included versus those excluded in this study.

Diagnosis of GDM was established by a 75-g oral glucose tolerance test (OGTT) at 24–28 weeks of gestation. GDM was diagnosed if the woman had two or more of the three plasma glucose values exceeding the following cutoffs: fasting, 5.3 mmol/L; 1 hour, 10.0 mmol/L; or 2 hour, 8.6 mmol/L (American Diabetes Association criteria) (18). There was no abnormal labor (e.g., prolonged labor or precipitate delivery) in every subject with or without GDM in this study. Every woman had been fasting since the beginning of the active phase in the first stage of labor. Exclusion criteria were: 1) a multiple pregnancy; 2) gestational age <37 weeks; 3) maternal age <18 or >45 years; 4) illicit drug use; 5) severe preexisting illnesses including pregestational diabetes mellitus, chronic hypertension, renal failure, pre-eclampsia, active or chronic liver diseases, epilepsy, serious pulmonary disease, serious hematological disorders, cancer, heart disease, or other life-threatening conditions; and 6) known fetal congenital anomalies or chromosomal abnormalities.

### Data and Blood Sample Collection

Maternal prepregnancy weight was obtained by face-to-face interview. Maternal weight close to delivery and height were measured in the hospital, and maternal body mass index (BMI) and weight gain were calculated. Neonatal birth weight and length were measured within one hour after delivery. Body mass index (BMI) and ponderal index (PI) were calculated: BMI = [weight (kilograms)/height (m)^2^], and PI = [birth weight (grams)/length (centimeters)^3^] ×100. Maternal fasting blood samples were collected about 1–3 days before delivery, at 37–41 weeks. Venous cord blood samples were obtained by syringe from the double-clamped venous cord immediately after delivery. Serum was separated by centrifugation and kept frozen in multiple aliquots at −80°C until analysis. Hemoglobin A1c levels were measured during the late pregnancy in the GDM group.

### Metabolic Parameters Assays

Glucose levels were assessed using Hitachi 7180 automated analyzer (Wako Diagnostics, Japan). Hemoglobin A1c levels were estimated using VARIANT II automated analyser (Bio-Rad, laboratories, Inc.). Insulin levels were measured using the electrochemistry immunoassay (ECL) method, with a COBAS e601 automated analyser (Roche Diagnostics, Germany). Proinsulin levels were determined by ELISA (R&D Systems, America), with an intra-assay CV of 5.6% and an inter-assay CV of 8.3%. The insulin resistance indexes were calculated according to the homeostasis model assessment of insulin resistance (HOMA-IR): (fasting serum insulin [microunits per milliliter] × fasting glucose [millimoles per liter])/22.5 [19].

### Statistical Analysis

Results were expressed as means ± SEM. Log transformation was applied for variables with skewed data distribution (insulin, proinsulin, HOMA-IR, and proinsulin-to-insulin ratio) in all statistical analysis. Differences between groups were examined with the Student’s t-test, and adjusted for potential confounders (maternal age, prepregnancy weight, and prepregnancy BMI), using univariate ANOVA analysis. The correlation analysis was tested with Pearson’s correlation analysis, and adjusted for potential confounders (maternal age and prepregnancy BMI), using partial correlations. All statistical analysis were performed with the software package SPSS version 17.0 (SPSS Inc., Chicago, IL, USA). A *P*-value <0.05 was considered statistically significant.

## Results

### Maternal and Pregnancy Characteristics

Maternal and fetal characteristics of total 148 Chinese pregnant women with or without GDM were shown in Table 1. The fetal condition was reflected by cord blood metabolic parameters. The pregnant women with GDM were slightly older, and had a higher prepregnancy weight and BMI than the control subjects. Maternal height, weight gain and Caesarean section rate did not differ between the two groups. Hemoglobin A1c level of GDM mothers was 5.29±0.06%, and in the mormal interval ranges. Neonatal birth weight, length and ponderal index were higher in the GDM group than the control group, although neonates in two groups were born at the similar gestational weeks.

**Table 1:** Maternal and fetal characteristics of pregnant women with or without GDM.

	Control	GDM	*P* value
**Subjects (n)**	83	65	
Maternal Age (years)	28.06±0.37	29.68±0.48	0.009
Maternal height (m)	161.25±0.48	161.05±0.53	NS
Prepregnancy weight (kg)	53.43±0.79	57.27±1.10	0.005
Prepregnancy BMI (kg/m^2^)	20.52±0.27	22.01±0.39	0.002
Maternal weight gain (kg)	13.62±0.34	14.76±0.66	NS
Gestational age (weeks)	39.02±0.13	38.78±0.11	NS
Caesarean section (% (*n*))	47.7% (31)	38.6% (32)	NS
Female sex, % (n)	52.3%(24)	50.6%(33)	NS
Birth weight (g)	3343±40	3581±58	0.001
Birth length (cm)	49.36±0.14	49.87±0.16	0.023
Ponderal index (g/cm^3^)	2.76±0.03	2.86±0.03	0.013

Data was expressed as means ± SEM.

a log-transformed skewed data were used for statistical comparisons.

b Maternal and fetal serum parameters were adjusted for maternal age, prepregnancy weight and prepregnancy BMI.

NS, not significant.

### Differences in Metabolic Parameters between the GDM and Control Groups

Both maternal and fetal levels of insulin, proinsulin and HOMA-IR were significantly higher in the GDM groups than the control subjects. Those differences were still significant, after adjustment for potential confounders (maternal age, prepregnancy weight and prepregnancy BMI) (Table 2). There’re no significant differences in maternal or fetal levels of glucose and proinsulin-to-insulin ratios between the GDM and control groups (Table 2).

**Table 2:** Maternal and fetal metabolic parameters of pregnant women with or without GDM.

	Control	GDM	*P* value	Adjusted *P* value^b^
**Maternal**				
Fasting glucose (mmol/L)	4.69±0.07	4.73±0.11	NS	NS
Insulin (µU/mL)^a^	15.38±1.19	24.79±1.70	<0.001	0.004
Proinsulin (pmol/L)^a^	16.17±1.84	23.30±2.05	0.005	0.005
HOMA-IR^a^	3.48±0.32	5.46±0.48	0.002	0.041
Proinsulin/insulin ratio (pmol/mU)^a^	1.36±0.21	1.28±0.13	NS	NS
**Fetal**				
Glucose (mmol/L)	3.89±0.05	4.08±0.08	NS	NS
Insulin (µU/mL)^a^	7.91±0.64	15.08±1.52	<0.001	<0.001
Proinsulin (pmol/L)^a^	15.07±1.23	25.82±2.92	0.017	0.015
HOMA-IR^a^	1.38±0.12	2.80±0.35	<0.001	0.017
Proinsulin/insulin ratio (pmol/mU)^a^	2.83±0.31	2.66±0.35	NS	NS

Data was expressed as means ± SEM.

a log-transformed skewed data were used for statistical comparisons.

b Maternal and fetal serum parameters were adjusted for maternal age, prepregnancy weight and prepregnancy BMI.

NS, not significant.

### Correlations between Fetal HOMA-IR and Maternal Metabolic Parameters

In the GDM group, fetal HOMA-IR was significantly correlated with maternal insulin, fasting glucose, HOMA-IR and proinsulin-to-insulin ratios (Table 2). Fetal proinsulin-to-insulin ratios were not significantly correlated with maternal HOMA-IR. After adjustment for maternal age and prepregnancy BMI, fetal HOMA-IR was significantly correlating with maternal HOMA-IR or insulin (adjusted, r = 0.307, *P = *0.019, and r = 0.261, *P* = 0.047, respectively) (Table 3). Similar correlations were observed in all the subjects with or without GDM (adjusted, r = 0.424, *P* = 0.008, and r = 0.415, *P* = 0.007, respectively). In the GDM group, neonatal ponderal index was significantly correlated with maternal or fetal HOMA-IR and insulin (Table 4). Those correlations remained significant after adjustment for maternal age and prepregnancy BMI (Table 4).

**Table 3:** Correlations between fetal HOMA-IR and metabolic parameters in pregnant women with GDM.

	Fetal HOMA-IR^a^			
	r	*P*	r^b^ (Adjusted)	*P* b (Adjusted )
**Maternal**				
HOMA-IR^a^	0.421	<0.001	0.307	0.019
Proinsulin/insulin(pmol/mU)^a^	−0.297	0.016	0.008	0.950
Insulin (µU/mL)^a^	0.378	0.002	0.261	0.047
Fasting glucose(mmol/L)	0.316	0.010	0.257	0.052

a log-transformed skewed data were used for statistical analysis.

b Partial correlations were adjusted for maternal age and prepregnancy BMI.

**Table 4:** Correlations between neonatal ponderal index and maternal or fetal metabolic parameters in pregnant women with GDM.

	Neonatal ponderalindex (g/cm^3^)			
	r	*P*	r^b^ (Adjusted)	*P* b (Adjusted)
MaternalHOMA-IR^a^	0.290	0.012	0.259	0.036
Maternal insulin(µU/mL)^a^	0.270	0.020	0.262	0.033
Fetal HOMA-IR^a^	0.471	<0.001	0.490	<0.001
Fetal insulin(µU/mL)^a^	0.510	<0.001	0.521	<0.001

a log-transformed skewed data were used for statistical analysis.

b Partial correlations were adjusted for maternal age and prepregnancy BMI.

## Discussion

Several studies on GDM have reported on the metabolic abnormalities of GDM mothers. Quite a few focused on maternal-fetal metabolic mechanisms in pregnant women with GDM.

This study tried to determine the effect of GDM on fetal insulin resistance or β-cell function in Chinese pregnant women with GDM. Our study reported i) fetal insulin resistance but not proinsulin-to-insulin ratios was significantly higher in Chinese pregnant women with GDM than control subjects, ii) fetal insulin resistance was correlated with maternal insulin resistance,iii) neonatal ponderal index was correlated with maternal or fetal insulin and insulin resistance.

In this study, we reported significantly higher levels of insulin, proinsulin and HOMA-IR, in Chinese pregnant women with GDM than control subjects, within 1–3 days before delivery, after adjustment for potential confounders. Similarly, we also reported significantly higher levels of insulin, proinsulin and HOMA-IR, in fetuses of Chinese pregnant women with GDM than control subjects, after adjustment for potential confounders. Such data clearly show a more highly insulin-resistant condition in fetuses of Chinese pregnant women with GDM than control subjects. Similar with this study of Chinese pregnant women, higher levels of insulin, proinsulin and HOMA-IR in fetuses of pregnant women with GDM were also reported in other different races and countries [17, 20, and 21].

However, even though both fetal levels of insulin and proinsulin levels were higher in GDM group, there’re no significant differences in fetal proinsulin-to-insulin ratios between the GDM and control groups. Such data agree with related fetal studies [17], and show no observably impaired β-cell function, in fetuses of GDM mothers than control subjects.

Several studies have focused on the effect of fetal growth on insulin resistance in infants, children, adolescents or adults [22]–[24]. While only a few latest data is available about the effects of maternal metabolic parameters on fetal insulin resistance. It was reported that there’s a strong positive correlation (r = 0.35) between maternal insulin resistance of obese mothers and fetal insulin resistance [16]. Similarly, it was reported that there’s a negative association between maternal OGTT blood glucose levels in mothers with GDM and fetal insulin sensitivity (r = −0.31) [17]. In accord with such data, this study reported that maternal insulin resistance significantly correlated with fetal insulin resistance in Chinese pregnant women with GDM (r = 0.307). As maternal insulin can not cross the placenta, GDM may thus affect fetal metabolic condition (such as insulin resistance) by maternal-fetal metabolic and/or epigenetic mechanisms. For example, a latest study from Salomón C et al. reported GDM reduces adenosine transport in human placental microvascular endothelium, by an effect of insulin [25]. Further research increasing that understanding may facilitate the development of potential effective interventions during GDM pregnancy, for breaking the mother-baby metabolic programming cycle.

As expected, neonatal ponderal index was significantly correlated with fetal levels of HOMA-IR and insulin in this study. We also reported significant correlations between neonatal ponderal index and maternal levels of HOMA-IR and insulin. In view of the associations of HOMA-IR or insulin levels between mothers and fetuses presented above, it was suggested that increased fetal insulin may play an important role in the GDM effect on increased neonatal fat mass.

The prevalence of GDM and macrosomia increased rapidly in Asia, especially in China. In view of Asian traditional custom of striving to ingest nutrition in gestation, this problem would become more and more seriously in future. It was suggested that glucose control may have attenuated the impact of GDM on fetal overgrowth in Chinese women in this study. Furthermore, our data presented higher fetal insulin resistance in Chinese pregnant women with GDM. As fetuses of a more highly insulin-resistant condition are more likely to develop metabolic syndromes and type II diabetes, this finding would be help to understand the mechanism in development and prevention of mother-baby diabetic cycle.

In summary, this study reported a higher fetal insulin resisitance in well-controlled Chinese pregnant women with GDM, which was correlated with maternal insulin resistance. Our findings demonstrate the effect of GDM on higher fetal insulin resistance, which may be help to present an opportunity for potential effective intervention during pregnancy on prevention of metabolic syndrome and type II diabetes.
